# Carnitine Deficiency in Chronic Kidney Disease: Pathophysiology, Clinical Implications, and Therapeutic Perspectives

**DOI:** 10.3390/nu17132084

**Published:** 2025-06-23

**Authors:** Yusuke Kaida, Kensei Taguchi, Kei Fukami

**Affiliations:** 1 Division of Nephrology, Department of Medicine, Kurume University School of Medicine, Kurume 830-0011, Japan; taguchi_kensei@med.kurume-u.ac.jp (K.T.); fukami@med.kurume-u.ac.jp (K.F.); 2 Research Institute of Medical Mass Spectrometry, Kurume University School of Medicine, Kurume 830-0011, Japan

**Keywords:** carnitine deficiency, chronic kidney disease (CKD), hemodialysis, L-carnitine supplementation

## Abstract

Carnitine is essential for the mitochondrial transport of long-chain fatty acids and thus plays a pivotal role in energy metabolism, particularly in metabolically active organs, such as skeletal and cardiac muscle. In patients with dialysis, carnitine homeostasis is disrupted because of the reduced synthesis, impaired renal reabsorption, and carnitine loss during extracorporeal procedures. Carnitine deficiency is linked to a wide range of clinical manifestations, including muscle weakness, treatment-resistant anemia, intradialytic hypotension, mental disorder, and cardiovascular disease. This review provides a comprehensive overview of the physiological function of carnitine, elucidates the underlying mechanisms of carnitine deficiency in patients with dialysis, and explores the clinical consequences. Furthermore, the efficacy and limitations of L-carnitine supplementation in clinical practice are discussed based on the current literature. A better understanding of the pathophysiological and clinical relevance of carnitine deficiency may help facilitate personalized therapeutic strategies for patients with kidney diseases.

## 1. Introduction

Carnitine plays a central role in cellular energy metabolism by facilitating the transport of long-chain fatty acids across the mitochondrial inner membrane to produce adenosine triphosphate (ATP) through β-oxidation. Not only is carnitine supplied exogenously through daily diet, but it is also endogenously synthesized in the liver, brain, and kidneys from two amino acids, lysine and methionine, to maintain a constant systemic availability [1,2,3]. Defects in carnitine homeostasis are observed often in patients with chronic kidney disease (CKD) undergoing hemodialysis (HD) [4,5]. Carnitine deficiency is caused by the dietary protein restrictions suggested by several guidelines for CKD [6,7], the impairment of carnitine reabsorption by kidney tubular cells, the reduced levels of carnitine synthesis, and extracorporeal carnitine loss during the procedure of HD and peritoneal dialysis (PD). Carnitine deficiency has been shown to be linked to a range of clinical manifestations, including muscle weakness and cramps, fatigue, erythropoietin-resistant anemia, and cardiac dysfunction, all of which are strongly associated with the reduced quality of life in patients with CKD [5,8,9]. Although the therapeutic benefits of L-carnitine supplementation have been demonstrated [10,11,12], clear consensus on its clinical indications, optimal dosing strategies, and long-term efficacy remains elusive; thus, the quality and consistency of the existing evidence continue to be subjects of debate. Given the emerging insights of carnitine deficiency on mitochondrial dysfunction and metabolic abnormalities in the progression of CKD, re-considering carnitine metabolism and physiological function and the clinical utility of L-carnitine supplementation would be of great importance. This review provides a comprehensive overview of carnitine metabolism and kinetics, elucidates the underlying mechanisms of carnitine deficiency relevant to CKD and dialysis, and discusses the current status and future perspectives of L-carnitine supplementation based on the latest evidence.

## 2. Physiological Roles and Metabolism of Carnitine

### 2.1. Structure of Carnitine

Carnitine is a low-molecular-weight substance that was first isolated in 1905 from extracts of bovine muscle tissue [1,13]. It was initially referred to as “vitamin B_T_” due to its essential role in the metamorphosis of *Tenebrio molitor* larvae into pupae. While vitamins are essential micronutrients that must be obtained from the diet owing to insufficient endogenous synthesis, carnitine is synthesized in the liver, brain, and kidney [2]. In contrast, carnitine possesses both a quaternary ammonium group (–N^+^(CH_3_)_3_) and a carboxylic acid group (–COOH), giving it an amino-acid-like structure, although it is not a constituent of proteins [1,2,3]. Therefore, carnitine is now classified as a vitamin-like compound. A wide range of physiological functions of carnitine have been discovered since the 1960s, with particular emphasis on its central role in fatty acid metabolism [14,15,16,17]. Carnitine is indispensable for the mitochondrial transport of long-chain fatty acids and for regulating the intracellular acetyl-CoA/CoA ratio, thereby acting as a key modulator of cellular energy homeostasis.

### 2.2. Role of Carnitine in Fatty Acid Metabolism

**Figure 1:**
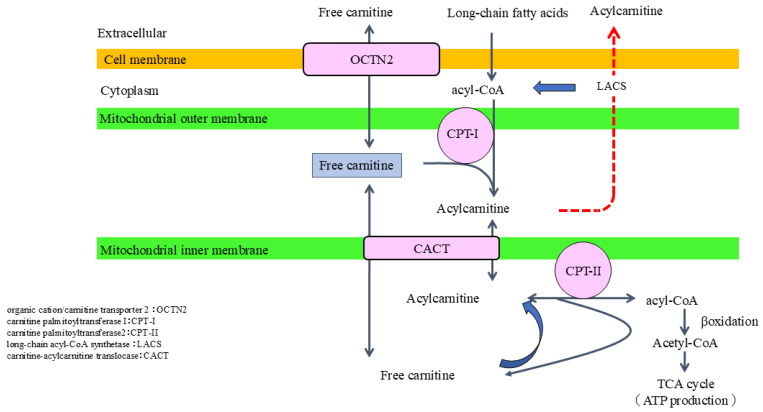
Carnitine shuttle system for fatty acid β-oxidation. Carnitine transports long-chain fatty acids into the mitochondrial matrix. Specifically, long-chain fatty acids are activated to acyl-CoA by LACS at the outer membrane of mitochondria. Acyl-CoA is then converted to acylcarnitine by CPT-1, and acylcarnitine is translocated across the mitochondrial inner membrane by CACT. Acylcarnitine is converted back to create acyl-CoA and free carnitine by CPT-2. The regenerated acyl-CoA undergoes β-oxidation producing acetyl-CoA, which enters TCA cycle to generate ATP. Circulating carnitine is absorbed by kidney tubular cells mainly through OCTN2. LACS, long-chain acyl-CoA synthetase; CPT-1, carnitine palmitoyltransferase 1; CACT, carnitine-acylcarnitine translocase; TCA, tricarboxylic acid; ATP, adenosine triphosphate; OCTN2, organic cation transporter 2.

Carnitine plays an essential role in fatty acid β-oxidation (FAO) to produce ATP as a central player in the “carnitine shuttle” system, which transports long-chain fatty acids into the mitochondrial matrix. Circulating carnitine is primarily absorbed through the main transporter organic cation/carnitine transporter 2 (OCTN2), which enables the maintenance of intracellular carnitine concentrations that substantially exceed those in the extracellular milieu [17,18]. Long-chain fatty acids are first activated to acyl-CoA by long-chain acyl-CoA synthetase (LACS), located on the outer mitochondrial membrane. The resulting acyl-CoA is then converted to acylcarnitine through the action of carnitine palmitoyltransferase 1 (CPT-1), also located on the outer mitochondrial membrane. Acylcarnitine is subsequently translocated across the inner mitochondrial membrane by carnitine-acylcarnitine translocase (CACT) and reconverted to acyl-CoA and free carnitine by CPT-II within the mitochondrial matrix. The regenerated acyl-CoA undergoes β-oxidation, producing acetyl-CoA, which enters the tricarboxylic acid (TCA) cycle, ultimately leading to ATP generation (Figure 1). Thus, carnitine is indispensable for the efficient metabolism of fatty acids and the maintenance of cellular energy homeostasis. Accordingly, absolute carnitine deficiency (i.e., decreased levels of free carnitine) or relative carnitine insufficiency (i.e., an elevated acylcarnitine-to-free carnitine ratio due to excess acyl-CoA accumulation) can impair the metabolic pathway, resulting in decreased ATP production [19,20].

### 2.3. Auxiliary Role of Carnitine in Metabolic Regulation

Regulation of the ratio of acyl-CoA to free CoA is another primary function of carnitine. The cellular pool of free CoA is limited and the reduced levels of free CoA impair the TCA cycle, urea cycle, gluconeogenesis, and glycolysis, resulting in mitochondrial dysfunction and disturbances in energy metabolism [17]. While this condition is known as CoA sequestration syndrome, which is pathogenesis for various metabolic disorders, carnitine helps to release free CoA from acyl-CoA by accepting acyl groups and transferring them as acylcarnitine. Carnitine-mediated “CoA rescue” mechanisms not only facilitate fatty acid metabolism but also support the proper functioning of the TCA cycle, urea cycle, and glycogenesis by preserving mitochondrial free CoA availability. From this point of view, carnitine deficiency has a huge impact on the energy metabolism of the cells [17,21,22].

Furthermore, carnitine is shown to exert pleiotropic cytoprotective effects against oxidative stress, inflammation, and apoptosis [23,24,25,26,27,28,29]. In addition to the above-mentioned effects of L-carnitine supplementation for proper mitochondrial respiration [30,31,32], L-carnitine activates and upregulates antioxidant enzymes, such as superoxide dismutase, catalase, and glutathione peroxidase [25]. Those actions mitigate lipid peroxidation, stabilize mitochondrial membranes, and produce other antioxidants, including glutathione and vitamin E, thereby maintaining redox status [30,31,32]. L-carnitine also activates the nuclear factor erythroid 2-related factor 2 (Nrf2) signaling, a central regulator of cellular antioxidant response, which enhances the expression of genes involved in detoxification and antioxidant defense via transcriptional activation [33,34,35].

Nuclear factor-κB (NF-κB) serves as a central hub in pro-inflammatory signaling [36], which is also known to be negatively regulated by carnitine. Thus, the impairment of carnitine homeostasis can promote the transcriptional activity of pro-inflammatory cytokines, including tissue necrotic factor-α, interleukin (IL)-6, and IL-1β [30,37]. Meanwhile, L-carnitine supplementation was shown to activate the anti-inflammatory transcription factor peroxisome proliferator-activated receptor gamma (PPAR-γ) and inhibit the phosphorylation of MAPK signaling pathways involved in cell proliferation and inflammation [38,39]. While excessive apoptosis, a programmed mechanism of cell death, establishes tissue damage [40], carnitine is known to inhibit apoptosis via stabilizing the mitochondrial membrane potential and then inhibiting the activation of caspase- and Bax-signaling [41]. Furthermore, carnitine modulates Toll-like receptor 4 signaling, which plays a critical role in the innate immune response, suggesting the immune-modulatory effects of carnitine [42,43]. Thus, both the physiological actions of carnitine and the carnitine-deficiency-related detrimental consequences have garnered increasing attention in several clinically significant conditions, such as Fanconi syndrome and cystinosis [44,45].

**Figure 2:**
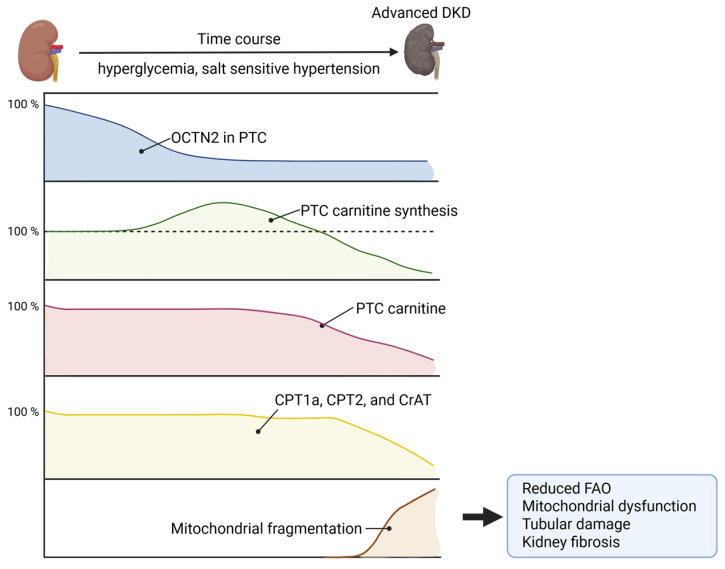
Time course toward advanced DKD. PTC OCTN2 expression is reduced, followed by compensatory upregulation of carnitine synthase enzymes. At a later stage, carnitine synthesis is reduced, which causes carnitine deficiency in PTC. Carnitine deficiency reduces carnitine transporter enzymes, such as CPT1a, CPT2, and CrAT, and contributes to mitochondrial fragmentation, leading to a reduction in fatty acid oxidation, mitochondrial dysfunction, tubular damage, and kidney fibrosis. These changes contribute to the progress of DKD. DKD, diabetic kidney disease; PTC, proximal tubular cells, CPT1a, carnitine palmitoyltransferase 1a; CPT2, carnitine palmitoyltransferase 2; CrAT, carnitine acetyltransferase; FAO, fatty acid oxidation; OCTN2, organic cation transporter 2.

Although the association between carnitine deficiency and mitochondrial dysfunction has been previously suggested, the underlying mechanisms and their contribution to the progression of CKD remain insufficiently understood. The latest basic research by Ito et al. demonstrated that expression of OCTN2, a main transporter responsible for carnitine absorption, in kidney tubular cells is reduced at the early stage of diabetic kidney disease (DKD), followed by comprehensive upregulation of carnitine synthesis. However, at a later stage, carnitine synthesis is impaired, which provokes carnitine deficiency, leading to the impairment of FAO and mitochondrial respiration. Those changes induce tubular damage and kidney fibrosis in the context of DKD. Furthermore, the reduction in kidney OCTN2 expression and resultant carnitine deficiency occurs in salt-sensitive hypertensive rats, suggesting that carnitine deficiency might be a common pathological pathway to promote kidney injury [46]. Meanwhile, L-carnitine supplementation alleviates kidney injury by improving fatty acid oxidation, mitochondrial respiration, and energy production in DKD and salt-sensitive rats. Carnitine palmitoyltransferase 1a (CPT1a) deficit is linked to advanced CKD [47], whereas the reduced CPT1a expression is attenuated by L-carnitine supplementation [46]; thus, restoration of carnitine-related FAO enzymes might become a potential therapeutic strategy to halt kidney injury (Figure 2).

### 2.4. Carnitine Synthesis and Metabolism

The carnitine pool consists of two major sources: dietary intake and endogenous synthesis [2,48]. A carnitine-rich diet includes red meat, fish, whole milk, and avocado, and dietary intake provides approximately 75% of the daily requirement in healthy adults. The remaining ~25% is supplied by endogenous synthesis, primarily in the liver and kidney, and to a lesser extent in the brain [2,48]. Carnitine biosynthesis begins with the essential amino acids lysine and methionine. The biosynthetic pathway involves a series of enzymatic reactions converting trimethyllysine into γ-butyrobetaine, and finally into carnitine [48,49,50]. This process requires several cofactors; for instance, vitamin C, niacin, vitamin B6, and iron are known to be involved, and thus, deficiency in any of these cofactors impairs carnitine synthesis. Among carnitine synthesis enzymes, γ-butyrobetaine dioxygenase, responsible for catalyzing the final conversion to carnitine, plays a rate-limiting role in the liver, kidney, and brain. Notably, the activity of carnitine biosynthesis enzymes increases with age in liver tissue: approximately 10% of adult levels at birth, 30% by age three, and reaching full adult levels around age fifteen [51,52,53]. By contrast, the enzyme activity in the kidney is equivalent to adult levels since the neonatal period, suggesting that the kidneys are the main source of carnitine during early life [52], but liver dysfunction due to metabolic dysfunction-associated steatotic liver disease, or cirrhosis and several kidney diseases might induce the decrease in carnitine synthesis, resulting in secondary carnitine deficiency [49,54].

Carnitine is predominantly distributed in skeletal muscle and cardiac muscle, which together account for approximately 98% of the total body storage. The estimated total carnitine pool is around 100 mmol in a 70 kg adult male and approximately 50 mmol in children [22,55,56]. Within those organs, carnitine levels are maintained 20–50 times higher than those in plasma [22,55]. Since approximately 0.6% of total carnitine exists in the plasma [55], it should be noted that plasma carnitine levels do not necessarily reflect organ storage levels of carnitine. Because the main carnitine storage is in skeletal muscles, individuals with reduced muscle mass, such as children, women, older adults, or patients with severe physical disabilities, sarcopenia, or cachexia, are more likely to develop carnitine deficiency [57,58]. The modern dietary environment also significantly influences carnitine homeostasis. Specifically, long-term reliance on enteral nutrition, total parenteral nutrition, or certain low-carnitine infant formulas causes carnitine deficiency [59,60]. Furthermore, it has been demonstrated that carnitine homeostasis heavily depends on renal reabsorption, excretion, and synthesis [3]. In the following section, we will introduce renal carnitine absorption and metabolic mechanisms, as well as the pathophysiological alterations associated with carnitine deficiency in CKD.

## 3. Impaired Carnitine Homeostasis in the Context of CKD

### 3.1. Impaired Endogenous Synthesis of Carnitine

The hydroxylation of γ-butyrobetaine to carnitine, the final step of carnitine synthesis, is catalyzed by γ-butyrobetaine dioxygenase (BBOX1). Recent studies have demonstrated a high expression of BBOX1 in kidney tissue, suggesting that the decline in renal function in CKD may downregulate BBOX1, which might cause reduced carnitine biosynthesis [61,62,63]. Moreover, chronic inflammation, oxidative stress, and protein-restricted diets for the comprehensive treatment of CKD may adversely affect both the availability and utilization of the amino acid precursors (lysine and methionine) and essential cofactors (e.g., vitamin C, niacin, vitamin B6, and iron) required for carnitine synthesis. In addition, carnitine is metabolized by gut microbiota to trimethylamine, which is oxidized in the liver by flavin-containing monooxygenase 3 to create trimethylamine-N-oxide (TMAO) [64]. CKD-related alterations in gut microbial composition, namely dysbiosis, are shown to promote the conversion of carnitine to TMA, indicating that available intestinal carnitine is expected to decline, possibly leading to carnitine deficiency in the body. Thus, patients with CKD are at high risk of developing carnitine deficiency via tubular damage, CKD-related inflammation, nutritional carnitine defects, and dysbiosis, and the carnitine deficiency might be a pathogenesis of CKD progression.

### 3.2. Insufficient Intake and Absorption Defects

Since the dietary regimen for patients with CKD is typically based on protein restriction to reduce intraglomerular pressure and minimize uremic toxin accumulation, the intake of carnitine-rich animal products, such as red meat, fish, whole milk, and avocado, is restricted. As a result, the amount of carnitine consumption is significantly reduced compared to that in healthy individuals [65,66]. Furthermore, with the progression of CKD, uremic toxin accumulation and longitudinal inflammation cause taste disturbances, resulting in appetite loss and malnutrition [67,68,69]. Furthermore, intestinal edema, inflammation, and uremic toxin accumulation in intestinal mucosa decrease OCTN2 expression and function in the context of CKD [70]. Thus, the above-mentioned multi-factors are involved in potential and subclinical carnitine deficiency.

### 3.3. Renal Excretion and Reabsorption of Carnitine

A large amount of carnitine is filtered through the glomerulus and excreted in the urine, but more than 90% is reabsorbed in kidney tubular cells. Excretion into the feces accounts for less than 1% of total carnitine excretion. The concentration of free carnitine, a non-protein-bound carnitine, is dependent on renal reabsorption owing to its low molecular weight, whereas acyl-carnitine is easily lost in the urine [55,71]. Therefore, tubular-damage-induced impairment of free carnitine reabsorption might provoke an impact on carnitine homeostasis [72]. In addition, at least 138 genetic variations in the OCTN2 gene have been reported so far [20] and its loss of function leads to carnitine deficiency [73,74]. Furthermore, several therapeutic compounds, such as valproic acid, cyclosporine, and cisplatin, are reported to inhibit the function of OCTN2, resulting in secondary carnitine deficiency [75,76,77,78,79]. Not only in the intestine but also in the kidney, the decreased OCTN2 due to pro-inflammatory cytokines and uremic toxin accumulation might be involved in potential carnitine deficiency [30,70].

### 3.4. Increased Loss of Carnitine During Hemodialysis Process

In patients undergoing HD, a rapid and significant decrease in plasma carnitine levels is observed after the initiation of HD treatment [80]. The HD-related carnitine deficiency is mainly attributed to not only the decline in endogenous synthesis in atrophic kidney and nutritional disturbances but also removal by HD procedure. Since the molecular weight of carnitine is 161 Da, this allows it to pass through the dialysis membrane into the dialysate. Approximately 70–100 mg of carnitine is shown to be removed during an HD session, and long-term HD treatment likely causes a progressive decline in carnitine levels. Although less carnitine removal is observed in patients with PD when compared to those with HD, the decrease in free carnitine concentrations is still noted due to the decline in endogenous synthesis and the effects of dietary restrictions [81,82]. Additionally, children with either HD or PD are reported to develop carnitine deficiency [83,84]. Thus, patients with dialysis are at high risk of developing carnitine-deficiency-induced clinical symptoms, such as treatment-resistant anemia, severe muscle cramps, general fatigue, cognitive impairment, and mental disorder [85]. The next section discusses the clinical significance of carnitine deficiency and potential therapeutic interventions in patients with dialysis.

## 4. Clinical Implications of Carnitine Deficiency

### 4.1. ESA Resistance and Its Impact on Anemia Treatment

Anemia in patients with dialysis is challenging, which is more significant in patients with low responsiveness to erythropoiesis-stimulating agents (ESAs) [86]. However, L-carnitine supplementation has recently become a potential therapeutic option for ESA-resistant anemia. Carnitine deficiency impairs erythrocyte energy metabolism, leading to shortened red blood cell lifespan, membrane instability, and increased oxidative stress, all of which contribute to worsening renal anemia [87]. Thus, carnitine supplementation attenuates the response to ESA, resulting in the improvement of anemia, which has been proven by previous clinical studies [88,89]. For instance, oral administration of 500 mg L-carnitine daily for 3 months increased hematocrit value by more than 2%, with the improvement of the iron utilization rate [88]. Furthermore, the increase in hemoglobin at 12 and 24 months after intravenous administration of 1000 mg L-carnitine three times per week was observed in HD patients [89]. A meta-analysis including 18 randomized controlled trials (RCTs) carried out with 1090 participants confirmed that L-carnitine supplementation increased plasma free carnitine levels, reduced the erythropoietin resistance index, and decreased the required dose of ESA without affecting hemoglobin or hematocrit [10]. These results suggest that L-carnitine may improve the efficiency of ESA, but not directly affect hematopoietic capacity. In addition, there is a positive correlation between carnitine deficiency and ESA resistance, and carnitine supplementation has slight effects on ESA responsiveness in patients with PD as well [90,91]. Because of the small sample sizes in existing studies, further research will be required to establish a definitive consensus on the efficacy of L-carnitine supplementation for anemia in patients with PD [92,93]. However, L-carnitine supplementation is already considered an adjunctive therapeutic intervention for anemia in patients with dialysis.

### 4.2. Cardiovascular Effects

There has been growing attention on the effects of L-carnitine supplementation on the cardiovascular system [94]. Carnitine regulates the transportation of fatty acids into mitochondria, which is particularly important in the heart because fatty acids are the primary energy source of ATP in cardiomyocytes [95]. Given that carnitine deficiency adversely affects cardiac function, it remains unclear whether L-carnitine supplementation can prevent or improve cardiovascular diseases. Higuchi et al. conducted RCT with patients with HD and demonstrated that administration of 20 mg/kg per day of oral L-carnitine for 12 months attenuated left ventricular ejection fraction by 5.43% from baseline. Furthermore, left ventricular mass index (LVMI), a sensitive marker for cardiac hypertrophy, decreased by 8.89 g/m^2^ in the L-carnitine-treated patients, while it increased by 1.62 g/m^2^ in the control group. Additionally, the N-terminal prohormone of brain natriuretic peptide (NT-proBNP), a marker for heart failure, was reduced by L-carnitine supplementation, suggesting that L-carnitine supplementation has a protective effect on cardiac function [96]. Similarly, another clinical study revealed an improvement in LVMI by L-carnitine supplementation, supporting the hypothesis regarding the protective and preventative efficacy of L-carnitine against heart failure and hypertrophy [97]. Kazmi et al. found that L-carnitine supplementation reduced the length of hospitalization in dialysis patients with angina, myocardial infarction, arrhythmias, congestive heart failure, cerebrovascular diseases, and peripheral arterial diseases [98]. Additionally, oral administration with 20 mg/kg per day of L-carnitine reduced the brachial ankle pulse wave velocity in patients with HD, which may be linked to suppressing atherosclerosis and cardiovascular disease [99]. TMAO is not only a biomarker of cardiovascular disease but also contributes directly to its pathogenesis by promoting endothelial cell injury and atherosclerosis [100,101]. In the gut, dietary carnitine is metabolized by the gut microbiota to trimethylamine, which, in turn, is oxidized to TMAO in the liver. Thus, oral administration with L-carnitine, but not the intravenous route, significantly increases serum TMAO levels [102], which is linked to induced atherosclerosis despite the antioxidant and anti-inflammatory properties of L-carnitine [103]. These observations and discrepancies underscore the need for further investigation to determine whether the route of L-carnitine administration differentially influences its cardiovascular effects, particularly in relation to endothelial function and atherosclerotic lesions.

### 4.3. Impact on Quality of Life

In patients with long-term dialysis, carnitine deficiency causes muscle symptoms, mental disorder, hemodynamic alterations during dialysis, and reduced QOL [104]. Given that the main role of carnitine is to facilitate β-oxidation, its deficiency is associated with various clinical symptoms, including chronic fatigue, muscle weakness, mental lethargy, and hypotension during dialysis. Siami et al. reported significant improvement in muscle strength in 9 out of 14 patients after intravenous administration of L-carnitine [105]. In an RCT, L-carnitine supplementation increased muscle mass and exercise tolerance (maximum oxygen uptake) [106]. Furthermore, the intravenous administration of L-carnitine was effective in maintaining upper arm muscle mass and grip strength [107]. The effects of L-carnitine supplementation are shown to be superior to those of cycle ergometer exercises widely used during the HD session [108]. Several clinical studies have reported preventive effects on muscle cramps, and the improvement in muscle-related symptoms is linked to attenuating QOL in patients with dialysis [8,12,106]. Beyond muscle symptoms, L-carnitine supplementation is known to prevent a rapid decrease in blood pressure as a result of ultrafiltration in dialysis, contributing to hemodynamic stabilization. In fact, L-carnitine administration reduced the incidence of hypotension during a dialysis session [8,106,109].

In addition to evidence from RCTs, several observational and retrospective studies have reported the clinical benefits of L-carnitine supplementation in dialysis patients, particularly in terms of symptom relief, such as reduced muscle cramps and better QOL [89,110,111]. HD patients with L-carnitine treatment sustained skeletal muscle mass and improved muscle strength and physical activity when compared to those with cycle ergometer exercise alone [108]. While patients with HD likely impair cardiac function, a top cause of mortality, L-carnitine supplementation was confirmed to improve cardiac function, particularly left ventricular ejection fraction. Furthermore, L-carnitine supplementation improved mental status via increasing testosterone in male patients with HD [112]. These findings enhance the practical relevance of its therapeutic strategies and support the individualized treatment decision, improving patients’ satisfaction and medication adherence.

HD patients who received oral L-carnitine supplementation for six months initially showed improvements in “general health” and “physical function”; however, the positive effects were not sustained over the six-month period [113]. Thus, the effectiveness of L-carnitine supplementation may vary depending on patient characteristics. Given the relatively well-established safety profile of L-carnitine supplementation, discontinuation may not be necessary in the absence of evident adverse effects. However, due to the lack of a consistent correlation between the severity of carnitine deficiency and the appearance of clinical symptoms, further research will be needed to clarify which populations obtain the most benefits from L-carnitine supplementation.

### 4.4. Impact on Clinical Parameters

Carnitine deficiency is closely associated with dyslipidemia, chronic inflammation, and malnutrition in patients with dialysis. Meta-analyses have suggested that L-carnitine supplementation might affect the levels of low-density lipoprotein (LDL) cholesterol, while no significant changes in triglycerides or high-density lipoprotein cholesterol were observed [114]. However, many of the interventional studies so far have involved small sample sizes or demonstrated limited effects. Some clinical research revealed an elevation in LDL cholesterol by L-carnitine treatment [115,116]. It should be noted that methodological heterogeneity among previous clinical studies, such as the administration route of L-carnitine, participants’ background, comorbidities, and dialysis vintage, might be associated with inconsistent results in some clinical parameters. For instance, as mentioned above, some research showed a reduction in LDL by L-carnitine, while others demonstrated increased LDL levels following the initiation of L-carnitine treatment in patients with HD. By contrast, it has been consistently proven that L-carnitine administration improved inflammatory status, such as decreased C-reactive protein and transferrin levels, which possibly leads to increased levels of LDL because chronic inflammation suppresses lipoprotein production in the liver. Similarly, L-carnitine administration attenuated malnutrition status in patients with HD, while malnutrition is linked to low LDL levels, suggesting that L-carnitine-induced improvement of nutrition status might be associated with an increase in LDL levels. Thus, multiple factors have a huge impact on lipid profiles, which might become statistical bias, causing several discrepancies in the results among previous clinical studies.

Furthermore, much attention has been given to the effects of L-carnitine on nutrition status. L-carnitine supplementation increased serum levels of albumin, total protein, transferrin, and pre-albumin, with an improvement in body mass index in patients with dialysis [11,117]. There is also accumulating evidence regarding the impact on inflammatory markers. A meta-analysis demonstrated a significant reduction in C-reactive protein (CRP) by L-carnitine supplementation [118,119]. Moreover, the effect of L-carnitine supplementation on uremic toxins has been shown. Fukami et al. demonstrated that intravenous administration at 900 mg L-carnitine per day for six months reduced the levels of advanced glycation endproducts (AGEs), a representative uremic toxin, in 102 patients with HD [120]. Also, L-carnitine supplementation was shown to increase testosterone, which is the primary male sex hormone that plays a crucial role in male mental health in patients with HD; thus, mental status was significantly improved by L-carnitine supplementation [112,121]. In summary, the pleiotropic efficacy of L-carnitine supplementation highlights its clinical importance in patients with dialysis.

### 4.5. Meta-Analysis of L-Carnitine Supplementation in Patients with Dialysis

Six meta-analyses so far have shown the benefits of L-carnitine supplementation in patients with dialysis (Table 1). The results from those RCTs indicate that L-carnitine supplementation offers certain therapeutic advantages in the following areas: (1) improvement in nutritional status; (2) potential modulation of lipid profiles; (3) reduction in muscle cramps; (4) decrease in inflammatory markers, particularly CRP value. L-carnitine supplementation has been proposed as a supportive treatment for improving malnutrition, muscle-related symptoms, and chronic inflammation—conditions frequently observed in patients with dialysis. Meanwhile, the effects on hemoglobin levels, blood pressure, and the required dosage of ESA were inconsistent across clinical studies, and current evidence remains insufficient to establish a definitive consensus. Large-scale and high-quality RCTs might be required to clarify the efficacy of L-carnitine on those parameters. Moreover, the influence of L-carnitine on cardiovascular events has not been fully explored and warrants further investigation. Regarding its safety concerns, previous studies have reported few serious adverse events associated with L-carnitine supplementation, supporting its favorable safety profile [114]. A 10-year follow-up carried out with patients with primary carnitine deficiency who have been taking carnitine supplementation revealed that L-carnitine supplement prevents cardiac complications, including cardiomyopathy, without major adverse effects, suggesting the safety of long-term L-carnitine administration [122]. Although no similar follow-up trial was carried out in patients with dialysis, no warnings regarding adverse effects or recommendations for discontinuation of L-carnitine have been issued by pharmaceutical companies or government agencies. These facts indicate the long-term safety of the use of L-carnitine in patients with dialysis. While the above-mentioned efficacy of L-carnitine administration has been reported, methodological heterogeneity exists in terms of dosage; method of administration (oral or intravenous); patients’ background, such as age, comorbidities, and dialysis history; and different primary endpoints. These differences may affect the consistency and reproducibility of the therapeutic effects of L-carnitine supplementation. Thus, future research should stratify the method, underlying diseases, dialysis vintage, and nutritional status for a better understanding of the effects of L-carnitine. In addition, accumulating real-world observational studies are expected to identify the patient populations most likely to benefit from carnitine supplementation and refine optimal treatment strategies.

## 5. Conclusions

Carnitine deficiency has been associated with a broad spectrum of symptoms, including muscle weakness, fatigue, muscle cramps, and subsequent reduced QOL, all of which may negatively influence treatment satisfaction and patient outcomes. Despite the established efficacy of L-carnitine supplementation, further investigations are warranted, particularly in the identification of appropriate patient populations and standardization of treatment algorithms for carnitine supplementation. Furthermore, large-scale, well-designed prospective studies and RCTs are required to assess long-term outcomes. Addressing these challenges and generating high-quality evidence will facilitate the establishment of L-carnitine supplementation as a standard adjunctive therapy in the management of CKD.

## Figures and Tables

**Table 1:** Summary of meta-analyses on L-carnitine supplementation in dialysis patients.

Author	Objective	Assessed Effects	Key Findings	Conclusion
Chewcharat et al., 2022 [12]	To evaluate the effect of oral or IV levocarnitine on dialysis-related hypotension and muscle cramps in HD patients	Dialysis-related hypotension, muscle cramps	↓ Hypotension (OR = 0.26); ↓ Muscle cramps (OR = 0.22); Oral route > IV; Effective dose > 4200 mg/week, duration ≥ 12 weeks	Oral L-carnitine ≥ 12 weeks reduces dialysis-related hypotension; may reduce muscle cramps.
Zhu et al., 2021 [10]	To evaluate the effect of L-carnitine supplementation on renal anemia in HD patients	Plasma L-carnitine levels, ESA dose, ERI, Hb, Ht	↑ Plasma free L-carnitine; ↓ ESA dose and ERI; No significant change in Hb and Ht	L-carnitine improves ESA responsiveness and reduces ESA dose but does not raise Hb or Ht.
Zhou et al., 2020 [11]	To evaluate the efficacy of L-carnitine in improving malnutrition in HD patients	Albumin, prealbumin, total protein, transferrin	↑ Albumin (SMD: 2.51); ↑ Prealbumin (MD: 70.86); ↑ Total protein (MD: 3.83); ↑ Transferrin (MD: 0.35); all *p* < 0.001	L-carnitine significantly improves nutritional biomarkers; it may be a beneficial treatment option for malnutrition
Chen et al., 2014 [114]	To reevaluate the effects of L-carnitine in adults with ESKD on maintenance HD	LDL, CRP, triglycerides, cholesterol, HDL, Hb, Ht, albumin, erythropoietin dose	L-carnitine significantly decreased LDL (MD: −5.82 mg/dL) and CRP (MD: −3.65 mg/L). No significant changes in triglycerides, cholesterol, HDL, hemoglobin, hematocrit, albumin, or erythropoietin dose	The study confirmed a significant reduction in CRP and LDL with L-carnitine, although only the CRP reduction may be clinically meaningful. No effect on anemia parameters or erythropoietin requirement was found.
Huang et al., 2013 [5]	To assess the effect of L-carnitine supplementation on serum lipid profile in HD patients	Total cholesterol, HDL, LDL, VLDL, triglycerides	No significant effects on total cholesterol (SMD −0.11), HDL (SMD 0.01), VLDL (SMD 0.54), or triglycerides (SMD −0.12); significant decrease in LDL (SMD −0.29; 95% CI −0.53 to −0.06)	L-carnitine significantly reduced LDL-cholesterol levels in HD patients, especially in those receiving intravenous administration and with longer treatment duration. No effect was observed on other lipid parameters.
Lynch et al., 2008 [8]	To assess the effects of L-carnitine supplementation on dialysis-related hypotension and muscle cramps in HD patients	Intradialytic muscle cramps and hypotension	L-carnitine reduced the odds of muscle cramps (OR: 0.30; 95% CI: 0.09–1.00; *p* = 0.05). No significant effect on hypotension (OR: 0.28; 95% CI: 0.04–2.23; *p* = 0.2)	Evidence suggests a potential benefit for muscle cramping, but not confirmed. No clear benefit for hypotension.

CI: confidence interval, CRP: C-reactive protein, ESA: erythropoiesis-stimulating agent, ERI: Erythropoiesis Resistance Index, Hb: hemoglobin, HD: hemodialysis, HDL: high-density lipoprotein, Ht: hematocrit, IV: intravenous injection, LDL: low-density lipoprotein, MD: mean difference, OR: odds ratio, SMD: standardized mean difference, VLDL: very low-density lipoprotein. The downward arrows (↓) under “Hypotension (OR = 0.26)” and “Muscle cramps (OR = 0.22)” indicate a decrease in the incidence of these conditions. The upward arrow (↑) for “Plasma free L-carnitine” indicates an increase in its levels, while the downward arrows (↓) for “ESA dose” and “ERI” indicate decreases. Similarly, the upward arrows (↑) for “Albumin (SMD: 2.51)”, “Prealbumin (MD: 70.86)”, “Total protein (MD: 3.83)”, and “Transferrin (MD: 0.35)” indicate increases in all these parameters.

## References

[1] Bremer J. (1983). Carnitine–metabolism and functions. Physiol. Rev..

[2] Rebouche C.J., Seim H. (1998). Carnitine metabolism and its regulation in microorganisms and mammals. Annu. Rev. Nutr..

[3] Rebouche C.J. (2004). Kinetics, pharmacokinetics, and regulation of L-carnitine and acetyl-L-carnitine metabolism. Ann. N. Y. Acad. Sci..

[4] Hurot J.M., Cucherat M., Haugh M., Fouque D. (2002). Effects of L-carnitine supplementation in maintenance hemodialysis patients: A systematic review. J. Am. Soc. Nephrol..

[5] Huang H., Song L., Zhang H., Zhang J., Zhao W. (2013). Influence of L-carnitine supplementation on serum lipid profile in hemodialysis patients: A systematic review and meta-analysis. Kidney Blood Press. Res..

[6] Ikizler T.A., Burrowes J.D., Byham-Gray L.D., Campbell K.L., Carrero J.J., Chan W., Fouque D., Friedman A.N., Ghaddar S., Goldstein-Fuchs D.J. (2020). KDOQI Clinical Practice Guideline for Nutrition in CKD: 2020 Update. Am. J. Kidney Dis..

[7] Kidney Disease: Improving Global Outcomes (KDIGO) CKD Work Group (2024). KDIGO 2024 Clinical Practice Guideline for the Evaluation and Management of Chronic Kidney Disease. Kidney Int..

[8] Lynch K.E., Feldman H.I., Berlin J.A., Flory J., Rowan C.G., Brunelli S.M. (2008). Effects of L-carnitine on dialysis-related hypotension and muscle cramps: A meta-analysis. Am. J. Kidney Dis..

[9] Calvani M., Benatti P., Mancinelli A., D’Iddio S., Giordano V., Koverech A., Amato A., Brass E.P. (2004). Carnitine replacement in end-stage renal disease and hemodialysis. Ann. N. Y. Acad. Sci..

[10] Zhu Y., Xue C., Ou J., Xie Z., Deng J. (2021). Effect of L-carnitine supplementation on renal anemia in patients on hemodialysis: A meta-analysis. Int. Urol. Nephrol..

[11] Zhou J., Yang T. (2020). The efficacy of L-carnitine in improving malnutrition in patients on maintenance hemodialysis: A meta-analysis. Biosci. Rep..

[12] Chewcharat A., Chewcharat P., Liu W., Cellini J., Phipps E.A., Melendez Young J.A., Nigwekar S.U. (2022). The effect of levocarnitine supplementation on dialysis-related hypotension: A systematic review, meta-analysis, and trial sequential analysis. PLoS ONE.

[13] Wolf G. (2006). The discovery of a vitamin role for carnitine: The first 50 years. J. Nutr..

[14] Angelini C., Vergani L., Martinuzzi A. (1992). Clinical and biochemical aspects of carnitine deficiency and insufficiency: Transport defects and inborn errors of beta-oxidation. Crit. Rev. Clin. Lab. Sci..

[15] Tein I. (2002). Role of carnitine and fatty acid oxidation and its defects in infantile epilepsy. J. Child. Neurol..

[16] Ramsay R.R., Zammit V.A. (2004). Carnitine acyltransferases and their influence on CoA pools in health and disease. Mol. Aspects Med..

[17] Hoppel C. (2003). The role of carnitine in normal and altered fatty acid metabolism. Am. J. Kidney Dis..

[18] Foster D.W. (2004). The role of the carnitine system in human metabolism. Ann. N. Y. Acad. Sci..

[19] Stanley C.A. (2004). Carnitine deficiency disorders in children. Ann. N. Y. Acad. Sci..

[20] Longo N., Frigeni M., Pasquali M. (2016). Carnitine transport and fatty acid oxidation. Biochim. Biophys. Acta.

[21] Pochini L., Scalise M., Galluccio M., Amelio L., Indiveri C. (2011). Reconstitution in liposomes of the functionally active human OCTN1 (SLC22A4) transporter overexpressed in Escherichia coli. Biochem. J..

[22] Stanley C.A. (1987). New genetic defects in mitochondrial fatty acid oxidation and carnitine deficiency. Adv. Pediatr..

[23] Borum P.R. (1983). Carnitine. Annu. Rev. Nutr..

[24] Hagen T.M., Moreau R., Suh J.H., Visioli F. (2002). Mitochondrial decay in the aging rat heart: Evidence for improvement by dietary supplementation with acetyl-L-carnitine and/or lipoic acid. Ann. N. Y. Acad. Sci..

[25] Cao Y., Qu H.J., Li P., Wang C.B., Wang L.X., Han Z.W. (2011). Single dose administration of L-carnitine improves antioxidant activities in healthy subjects. Tohoku J. Exp. Med..

[26] Lee B.J., Lin J.S., Lin Y.C., Lin P.T. (2014). Effects of L-carnitine supplementation on oxidative stress and antioxidant enzymes activities in patients with coronary artery disease: A randomized, placebo-controlled trial. Nutr. J..

[27] Arockia Rani P.J., Panneerselvam C. (2001). Carnitine as a free radical scavenger in aging. Exp. Gerontol..

[28] Vardiyan R., Ezati D., Anvari M., Ghasemi N., Talebi A. (2020). Effect of L-carnitine on the expression of the apoptotic genes Bcl-2 and Bax. Clin. Exp. Reprod. Med..

[29] Lee Y.G., Chou H.C., Chen Y.T., Tung S.Y., Ko T.L., Buyandelger B., Wen L.L., Juan S.H. (2022). L-Carnitine reduces reactive oxygen species/endoplasmic reticulum stress and maintains mitochondrial function during autophagy-mediated cell apoptosis in perfluorooctanesulfonate-treated renal tubular cells. Sci. Rep..

[30] Xiang F., Zhang Z., Xie J., Xiong S., Yang C., Liao D., Xia B., Lin L. (2025). Comprehensive review of the expanding roles of the carnitine pool in metabolic physiology: Beyond fatty acid oxidation. J. Transl. Med..

[31] Virmani M.A., Cirulli M. (2022). The Role of l-Carnitine in Mitochondria, Prevention of Metabolic Inflexibility and Disease Initiation. Int. J. Mol. Sci..

[32] Sharma S., Black S.M. (2009). Carnitine homeostasis, mitochondrial function, and cardiovascular disease. Drug Discov. Today Dis. Mech..

[33] Li J., Zhang Y., Luan H., Chen X., Han Y., Wang C. (2016). l-carnitine protects human hepatocytes from oxidative stress-induced toxicity through Akt-mediated activation of Nrf2 signaling pathway. Can. J. Physiol. Pharmacol..

[34] Calabrese V., Cornelius C., Mancuso C., Pennisi G., Calafato S., Bellia F., Bates T.E., Giuffrida Stella A.M., Schapira T., Dinkova Kostova A.T. (2008). Cellular stress response: A novel target for chemoprevention and nutritional neuroprotection in aging, neurodegenerative disorders and longevity. Neurochem. Res..

[35] Yang T., Liu S., Ma H., Lai H., Wang C., Ni K., Lu Y., Li W., Hu X., Zhou Z. (2024). Carnitine functions as an enhancer of NRF2 to inhibit osteoclastogenesis via regulating macrophage polarization in osteoporosis. Free Radic. Biol. Med..

[36] Ghosh S., Karin M. (2002). Missing pieces in the NF-kappaB puzzle. Cell.

[37] Mahfouz R., H El-Rewini S., I Ghoneim A., Sheta E., A Ali M., Ibrahim S.S.A. (2024). L-Carnitine augments probenecid anti-inflammatory effect in monoiodoacetate-induced knee osteoarthritis in rats: Involvement of miRNA-373/P2X7/NLRP3/NF-κB milieu. Inflammopharmacology.

[38] Zambrano S., Blanca A.J., Ruiz-Armenta M.V., Miguel-Carrasco J.L., Arévalo M., Vázquez M.J., Mate A., Vázquez C.M. (2013). L-Carnitine protects against arterial hypertension-related cardiac fibrosis through modulation of PPAR-γ expression. Biochem. Pharmacol..

[39] Mansour H.H., El Kiki S.M., Ibrahim A.B., Omran M.M. (2021). Effect of l-carnitine on cardiotoxicity and apoptosis induced by imatinib through PDGF/PPARγ/MAPK pathways. Arch. Biochem. Biophys..

[40] Taguchi K., Sugahara S., Elias B.C., Pabla N.S., Canaud G., Brooks C.R. (2024). IL-22 is secreted by proximal tubule cells and regulates DNA damage response and cell death in acute kidney injury. Kidney Int..

[41] Koohpeyma F., Siri M., Allahyari S., Mahmoodi M., Saki F., Dastghaib S. (2021). The effects of L-carnitine on renal function and gene expression of caspase-9 and Bcl-2 in monosodium glutamate-induced rats. BMC Nephrol..

[42] Sun C., Guo Y., Cong P., Tian Y., Gao X. (2023). Liver Lipidomics Analysis Revealed the Novel Ameliorative Mechanisms of L-Carnitine on High-Fat Diet-Induced NAFLD Mice. Nutrients.

[43] Jamali-Raeufy N., Alizadeh F., Mehrabi Z., Mehrabi S., Goudarzi M. (2021). Acetyl-L-carnitine confers neuroprotection against lipopolysaccharide (LPS) -induced neuroinflammation by targeting TLR4/NFκB, autophagy, inflammation and oxidative stress. Metab. Brain Dis..

[44] Steinmann B., Bachmann C., Colombo J.P., Gitzelmann R. (1987). The renal handling of carnitine in patients with selective tubulopathy and with Fanconi syndrome. Pediatr. Res..

[45] Gahl W.A., Bernardini I., Dalakas M., Rizzo W.B., Harper G.S., Hoeg J.M., Hurko O., Bernar J. (1988). Oral carnitine therapy in children with cystinosis and renal Fanconi syndrome. J. Clin. Invest..

[46] Ito S., Taguchi K., Kodama G., Kubo S., Moriyama T., Yamashita Y., Yokota Y., Nakayama Y., Kaida Y., Shinohara M. (2025). Involvement of impaired carnitine-induced fatty acid oxidation in experimental and human diabetic kidney disease. JCI Insight.

[47] Miguel V., Tituaña J., Herrero J.I., Herrero L., Serra D., Cuevas P., Barbas C., Puyol D.R., Márquez-Expósito L., Ruiz-Ortega M. (2021). Renal tubule Cpt1a overexpression protects from kidney fibrosis by restoring mitochondrial homeostasis. J. Clin. Invest..

[48] Steiber A., Kerner J., Hoppel C.L. (2004). Carnitine: A nutritional, biosynthetic, and functional perspective. Mol. Aspects Med..

[49] Krähenbühl S. (1996). Carnitine metabolism in chronic liver disease. Life Sci..

[50] Hoppel C.L., Davis A.T. (1986). Inter-tissue relationships in the synthesis and distribution of carnitine. Biochem. Soc. Trans..

[51] Rebouche C.J., Engel A.G. (1980). Tissue distribution of carnitine biosynthetic enzymes in man. Biochim. Biophys. Acta.

[52] Vaz F.M., Wanders R.J. (2002). Carnitine biosynthesis in mammals. Biochem. J..

[53] Olson A.L., Rebouche C.J. (1987). gamma-Butyrobetaine hydroxylase activity is not rate limiting for carnitine biosynthesis in the human infant. J. Nutr..

[54] Wanner C., Hörl W.H. (1988). Carnitine abnormalities in patients with renal insufficiency. Pathophysiological and therapeutical aspects. Nephron.

[55] Borum P.R., Bennett S.G. (1986). Carnitine as an essential nutrient. J. Am. Coll. Nutr..

[56] Rebouche C.J., Engel A.G. (1984). Kinetic compartmental analysis of carnitine metabolism in the human carnitine deficiency syndromes. Evidence for alterations in tissue carnitine transport. J. Clin. Invest..

[57] Crentsil V. (2010). Mechanistic contribution of carnitine deficiency to geriatric frailty. Ageing Res. Rev..

[58] Sawicka A.K., Hartmane D., Lipinska P., Wojtowicz E., Lysiak-Szydlowska W., Olek R.A. (2018). l-Carnitine Supplementation in Older Women. A Pilot Study on Aging Skeletal Muscle Mass and Function. Nutrients.

[59] Borum P.R. (2009). Carnitine in parenteral nutrition. Gastroenterology.

[60] Schmidt-Sommerfeld E., Penn D., Wolf H. (1983). Carnitine deficiency in premature infants receiving total parenteral nutrition: Effect of L-carnitine supplementation. J. Pediatr..

[61] Zhou L.T., Lv L.L., Qiu S., Yin Q., Li Z.L., Tang T.T., Ni L.H., Feng Y., Wang B., Ma K.L. (2019). Bioinformatics-based discovery of the urinary BBOX1 mRNA as a potential biomarker of diabetic kidney disease. J. Transl. Med..

[62] Liao C., Hu L., Jia L., Zhou J., Wang T., Kim K., Zhong H., Yao H., Dong L., Guo L. (2025). BBOX1 restrains TBK1-mTORC1 oncogenic signaling in clear cell renal cell carcinoma. Nat. Commun..

[63] Lindstedt G., Lindstedt S., Nordin I. (1982). Gamma-butyrobetaine hydroxylase in human kidney. Scand. J. Clin. Lab. Invest..

[64] Wang Z., Klipfell E., Bennett B.J., Koeth R., Levison B.S., Dugar B., Feldstein A.E., Britt E.B., Fu X., Chung Y.M. (2011). Gut flora metabolism of phosphatidylcholine promotes cardiovascular disease. Nature.

[65] Mafra D., Borges N.A., Cardozo L.F.M.F., Anjos J.S., Black A.P., Moraes C., Bergman P., Lindholm B., Stenvinkel P. (2018). Red meat intake in chronic kidney disease patients: Two sides of the coin. Nutrition.

[66] Fornasini G., Upton R.N., Evans A.M. (2007). A pharmacokinetic model for L-carnitine in patients receiving haemodialysis. Br. J. Clin. Pharmacol..

[67] Iorember F.M. (2018). Malnutrition in Chronic Kidney Disease. Front. Pediatr..

[68] Sguanci M., Ferrara G., Palomares S.M., Parozzi M., Godino L., Gazineo D., Anastasi G., Mancin S. (2024). Dysgeusia and Chronic Kidney Disease: A Scoping Review. J. Ren. Nutr..

[69] Rahbar Saadat Y., Abbasi A., Hejazian S.S., Hekmatshoar Y., Ardalan M., Farnood F., Zununi Vahed S. (2025). Combating chronic kidney disease-associated cachexia: A literature review of recent therapeutic approaches. BMC Nephrol..

[70] Isaka Y. (2017). Adaptor protein is a new therapeutic target in chronic kidney disease. Kidney Int..

[71] Engel A.G., Rebouche C.J., Wilson D.M., Glasgow A.M., Romshe C.A., Cruse R.P. (1981). Primary systemic carnitine deficiency. II. Renal handling of carnitine. Neurology.

[72] Bellinghieri G., Santoro D., Calvani M., Mallamace A., Savica V. (2003). Carnitine and hemodialysis. Am. J. Kidney Dis..

[73] Nezu J., Tamai I., Oku A., Ohashi R., Yabuuchi H., Hashimoto N., Nikaido H., Sai Y., Koizumi A., Shoji Y. (1999). Primary systemic carnitine deficiency is caused by mutations in a gene encoding sodium ion-dependent carnitine transporter. Nat. Genet..

[74] Koizumi A., Nozaki J., Ohura T., Kayo T., Wada Y., Nezu J., Ohashi R., Tamai I., Shoji Y., Takada G. (1999). Genetic epidemiology of the carnitine transporter OCTN2 gene in a Japanese population and phenotypic characterization in Japanese pedigrees with primary systemic carnitine deficiency. Hum. Mol. Genet..

[75] Raskind J.Y., El-Chaar G.M. (2000). The role of carnitine supplementation during valproic acid therapy. Ann. Pharmacother..

[76] Opala G., Winter S., Vance C., Vance H., Hutchison H.T., Linn L.S. (1991). The effect of valproic acid on plasma carnitine levels. Am. J. Dis. Child..

[77] Origlia N., Migliori M., Panichi V., Filippi C., Bertelli A., Carpi A., Giovannini L. (2006). Protective effect of L-propionylcarnitine in chronic cyclosporine-a induced nephrotoxicity. Biomed. Pharmacother..

[78] Ito T., Tsukahara K., Sato H., Shimizu A., Okamoto I. (2021). Changes in carnitine levels through induction chemotherapy in head and neck cancer patients as a potential cause of therapy-related malaise. BMC Cancer.

[79] Lancaster C.S., Hu C., Franke R.M., Filipski K.K., Orwick S.J., Chen Z., Zuo Z., Loos W.J., Sparreboom A. (2010). Cisplatin-induced downregulation of OCTN2 affects carnitine wasting. Clin. Cancer Res..

[80] Evans A. (2003). Dialysis-related carnitine disorder and levocarnitine pharmacology. Am. J. Kidney Dis..

[81] Guarnieri G., Biolo G., Vinci P., Massolino B., Barazzoni R. (2007). Advances in carnitine in chronic uremia. J. Ren. Nutr..

[82] Guarnieri G., Situlin R., Biolo G. (2001). Carnitine metabolism in uremia. Am. J. Kidney Dis..

[83] Aguilar-Kitsu A., Ibarra-Cazares P., Mendoza-Guevara L., Villasis-Keever M.A., Perez Andrade M.E., Castillo-Romero L., Morales-Nava A., Rodriguez-Leyva F., Sanchez-Barbosa L. (2006). Frequency of low carnitine levels in children on dialysis. Adv. Perit. Dial..

[84] Naseri M., Mottaghi Moghadam Shahri H., Horri M., Esmaeeli M., Ghaneh Sherbaf F., Jahanshahi S., Moeenolroayaa G., Rasoli Z., Salemian F., Pour Hasan M. (2016). Absolute and Relative Carnitine Deficiency in Patients on Hemodialysis and Peritoneal Dialysis. Iran. J. Kidney Dis..

[85] Calò L.A., Vertolli U., Davis P.A., Savica V. (2012). L carnitine in hemodialysis patients. Hemodial. Int..

[86] Akizawa T., Okumura H., Alexandre A.F., Fukushima A., Kiyabu G., Dorey J. (2018). Burden of Anemia in Chronic Kidney Disease Patients in Japan: A Literature Review. Ther. Apher. Dial..

[87] Bonomini M., Zammit V., Pusey C.D., De Vecchi A., Arduini A. (2011). Pharmacological use of L-carnitine in uremic anemia: Has its full potential been exploited?. Pharmacol. Res..

[88] Matsumoto Y., Amano I., Hirose S., Tsuruta Y., Hara S., Murata M., Imai T. (2001). Effects of L-carnitine supplementation on renal anemia in poor responders to erythropoietin. Blood Purif..

[89] Kuwasawa-Iwasaki M., Io H., Muto M., Ichikawa S., Wakabayashi K., Kanda R., Nakata J., Nohara N., Tomino Y., Suzuki Y. (2020). Effects of L-Carnitine Supplementation in Patients Receiving Hemodialysis or Peritoneal Dialysis. Nutrients.

[90] Kaneko S., Hirai K., Morino J., Minato S., Yanai K., Mutsuyoshi Y., Ishii H., Matsuyama M., Kitano T., Shindo M. (2020). Association between carnitine deficiency and the erythropoietin resistance index in patients undergoing peritoneal dialysis: A cross-sectional observational study. Ren. Fail..

[91] Kaneko S., Yanai K., Kitano T., Miyazawa H., Hirai K., Ookawara S., Morishita Y. (2021). Change in Anemia by Carnitine Supplementation in Patients Undergoing Peritoneal Dialysis: A Retrospective Observational Study. Front. Med..

[92] Verrina E., Caruso U., Calevo M.G., Emma F., Sorino P., De Palo T., Lavoratti G., Turrini Dertenois L., Cassanello M., Cerone R. (2007). Effect of carnitine supplementation on lipid profile and anemia in children on chronic dialysis. Pediatr. Nephrol..

[93] Lilien M.R., Duran M., Quak J.M., Frankhuisen J.J., Schröder C.H. (2000). Oral L-carnitine does not decrease erythropoietin requirement in pediatric dialysis. Pediatr. Nephrol..

[94] Pauly D.F., Pepine C.J. (2003). The role of carnitine in myocardial dysfunction. Am. J. Kidney Dis..

[95] van der Vusse G.J., van Bilsen M., Glatz J.F. (2000). Cardiac fatty acid uptake and transport in health and disease. Cardiovasc. Res..

[96] Higuchi T., Abe M., Yamazaki T., Okawa E., Ando H., Hotta S., Oikawa O., Kikuchi F., Okada K., Soma M. (2016). Levocarnitine Improves Cardiac Function in Hemodialysis Patients With Left Ventricular Hypertrophy: A Randomized Controlled Trial. Am. J. Kidney Dis..

[97] Sakurabayashi T., Miyazaki S., Yuasa Y., Sakai S., Suzuki M., Takahashi S., Hirasawa Y. (2008). L-carnitine supplementation decreases the left ventricular mass in patients undergoing hemodialysis. Circ. J..

[98] Kazmi W.H., Obrador G.T., Sternberg M., Lindberg J., Schreiber B., Lewis V., Pereira B.J. (2005). Carnitine therapy is associated with decreased hospital utilization among hemodialysis patients. Am. J. Nephrol..

[99] Higuchi T., Abe M., Yamazaki T., Mizuno M., Okawa E., Ando H., Oikawa O., Okada K., Kikuchi F., Soma M. (2014). Effects of levocarnitine on brachial-ankle pulse wave velocity in hemodialysis patients: A randomized controlled trial. Nutrients.

[100] Koeth R.A., Wang Z., Levison B.S., Buffa J.A., Org E., Sheehy B.T., Britt E.B., Fu X., Wu Y., Li L. (2013). Intestinal microbiota metabolism of L-carnitine, a nutrient in red meat, promotes atherosclerosis. Nat. Med..

[101] Miller M.J., Bostwick B.L., Kennedy A.D., Donti T.R., Sun Q., Sutton V.R., Elsea S.H. (2016). Chronic Oral L-Carnitine Supplementation Drives Marked Plasma TMAO Elevations in Patients with Organic Acidemias Despite Dietary Meat Restrictions. JIMD Rep..

[102] Ji W., Zhang B., Liu J., Li K., Jia J., Fan F., Jiang J., Wang X., Zhang Y. (2025). Relationship Between Level of Trimethylamine Oxide and the Risk of Recurrent Cardiovascular Events in Patients with Acute Myocardial Infarction. Nutrients.

[103] Fukami K., Yamagishi S., Sakai K., Kaida Y., Yokoro M., Ueda S., Wada Y., Takeuchi M., Shimizu M., Yamazaki H. (2015). Oral L-carnitine supplementation increases trimethylamine-N-oxide but reduces markers of vascular injury in hemodialysis patients. J. Cardiovasc. Pharmacol..

[104] Takashima H., Maruyama T., Abe M. (2021). Significance of Levocarnitine Treatment in Dialysis Patients. Nutrients.

[105] Siami G., Clinton M.E., Mrak R., Griffis J., Stone W. (1991). Evaluation of the effect of intravenous L-carnitine therapy on function, structure and fatty acid metabolism of skeletal muscle in patients receiving chronic hemodialysis. Nephron.

[106] Ahmad S., Robertson H.T., Golper T.A., Wolfson M., Kurtin P., Katz L.A., Hirschberg R., Nicora R., Ashbrook D.W., Kopple J.D. (1990). Multicenter trial of L-carnitine in maintenance hemodialysis patients. II. Clinical and biochemical effects. Kidney Int..

[107] Maruyama T., Maruyama N., Higuchi T., Nagura C., Takashima H., Kitai M., Utsunomiya K., Tei R., Furukawa T., Yamazaki T. (2019). Efficacy of L-carnitine supplementation for improving lean body mass and physical function in patients on hemodialysis: A randomized controlled trial. Eur. J. Clin. Nutr..

[108] Yano J., Kaida Y., Maeda T., Hashida R., Tonan T., Nagata S., Hazama T., Nakayama Y., Ito S., Kurokawa Y. (2021). l-carnitine supplementation vs cycle ergometer exercise for physical activity and muscle status in hemodialysis patients: A randomized clinical trial. Ther. Apher. Dial..

[109] Ibarra-Sifuentes H.R., Del Cueto-Aguilera Á., Gallegos-Arguijo D.A., Castillo-Torres S.A., Vera-Pineda R., Martínez-Granados R.J., Atilano-Díaz A., Cuellar-Monterrubio J.E., Pezina-Cantú C.O., Martínez-Guevara E.J. (2017). Levocarnitine Decreases Intradialytic Hypotension Episodes: A Randomized Controlled Trial. Ther. Apher. Dial..

[110] Rathod R., Baig M.S., Khandelwal P.N., Kulkarni S.G., Gade P.R., Siddiqui S. (2006). Results of a single blind, randomized, placebo-controlled clinical trial to study the effect of intravenous L-carnitine supplementation on health-related quality of life in Indian patients on maintenance hemodialysis. Indian J. Med. Sci..

[111] Sakurauchi Y., Matsumoto Y., Shinzato T., Takai I., Nakamura Y., Sato M., Nakai S., Miwa M., Morita H., Miwa T. (1998). Effects of L-carnitine supplementation on muscular symptoms in hemodialyzed patients. Am. J. Kidney Dis..

[112] Sakai K., Fukami K., Yamagishi S., Kaida Y., Adachi T., Ando R., Manabe R., Otsuka A., Sugi K., Ueda S. (2013). Evidence for a positive association between serum carnitine and free testosterone levels in uremic men with hemodialysis. Rejuvenation Res..

[113] Sloan R.S., Kastan B., Rice S.I., Sallee C.W., Yuenger N.J., Smith B., Ward R.A., Brier M.E., Golper T.A. (1998). Quality of life during and between hemodialysis treatments: Role of L-carnitine supplementation. Am. J. Kidney Dis..

[114] Chen Y., Abbate M., Tang L., Cai G., Gong Z., Wei R., Zhou J., Chen X. (2014). L-Carnitine supplementation for adults with end-stage kidney disease requiring maintenance hemodialysis: A systematic review and meta-analysis. Am. J. Clin. Nutr..

[115] Naini A.E., Sadeghi M., Mortazavi M., Moghadasi M., Harandi A.A. (2012). Oral carnitine supplementation for dyslipidemia in chronic hemodialysis patients. Saudi J. Kidney Dis. Transpl..

[116] Hamedi-Kalajahi F., Zarezadeh M., Mojtahedi S.Y., Shabbidar S., Fahimi D., Imani H. (2021). Effect of L-carnitine supplementation on lipid profile and apolipoproteins in children on hemodialysis: A randomized placebo-controlled clinical trial. Pediatr. Nephrol..

[117] Duranay M., Akay H., Yilmaz F.M., Senes M., Tekeli N., Yücel D. (2006). Effects of L-carnitine infusions on inflammatory and nutritional markers in haemodialysis patients. Nephrol. Dial. Transplant..

[118] Shakeri A., Tabibi H., Hedayati M. (2010). Effects of L-carnitine supplement on serum inflammatory cytokines, C-reactive protein, lipoprotein (a), and oxidative stress in hemodialysis patients with Lp (a) hyperlipoproteinemia. Hemodial. Int..

[119] Suchitra M.M., Ashalatha V.L., Sailaja E., Rao A.M., Reddy V.S., Bitla A.R., Sivakumar V., Rao P.V. (2011). The effect of L-carnitine supplementation on lipid parameters, inflammatory and nutritional markers in maintenance hemodialysis patients. Saudi J. Kidney Dis. Transpl..

[120] Fukami K., Yamagishi S., Sakai K., Kaida Y., Adachi T., Ando R., Okuda S. (2013). Potential inhibitory effects of L-carnitine supplementation on tissue advanced glycation end products in patients with hemodialysis. Rejuvenation Res..

[121] Tashiro K., Kaida Y., Yamagishi S.I., Tanaka H., Yokoro M., Yano J., Sakai K., Kurokawa Y., Taguchi K., Nakayama Y. (2017). L-Carnitine Supplementation Improves Self-Rating Depression Scale Scores in Uremic Male Patients Undergoing Hemodialysis. Lett. Drug Des. Discov..

[122] Abrahamsen R.K., Lund A.M., Rasmussen J. (2023). Patients with primary carnitine deficiency treated with L-carnitine are alive and doing well-A 10-year follow-up in the Faroe Islands. JIMD Rep..

